# Factors associated with knowledge and awareness of stroke in the Iraqi population: a cross-sectional study

**DOI:** 10.3389/fneur.2023.1144481

**Published:** 2023-04-18

**Authors:** Hala Al-Obaidi, Zainab Khidhair, Feras Jirjees, Muna Barakat, Husam AlSalamat, Zelal Kharaba, Yassen Alfoteih, Chadia Haddad, Sara Mansour, Souheil Hallit, Diana Malaeb, Hassan Hosseini

**Affiliations:** ^1^Department of Clinical Sciences, College of Pharmacy and Health Sciences, Ajman University, Ajman, United Arab Emirates; ^2^Department of Biology, College of Science, University of Baghdad, Baghdad, Iraq; ^3^Department of Pharmacy Practice and Pharmacotherapeutics, College of Pharmacy, University of Sharjah, Sharjah, United Arab Emirates; ^4^Department of Clinical Pharmacy and Therapeutics, Faculty of Pharmacy, Applied Science Private University, Amman, Jordan; ^5^MEU Research Unit, Middle East University, Amman, Jordan; ^6^Department of Basic Medical Sciences, Faculty of Medicine, Al-Balqa Applied University, Al-Salt, Jordan; ^7^Department of Biopharmaceutics and Clinical Pharmacy, School of Pharmacy, University of Jordan, Amman, Jordan; ^8^Department of Clinical Pharmacy, College of Pharmacy, Al-Ain University, Abu Dhabi, United Arab Emirates; ^9^Honorary Associate Lecturer, Faculty of Medical Sciences, Newcastle University, Newcastle upon Tyne, United Kingdom; ^10^Department of Dental Surgery, City University College of Ajman, Ajman, United Arab Emirates; ^11^INSPECT-LB (Institut National de Santé Publique, d'Épidémiologie Clinique et de Toxicologie-Liban), Beirut, Lebanon; ^12^Research Department, Psychiatric Hospital of the Cross, Jal Ed Dib, Lebanon; ^13^School of Pharmacy, Lebanese International University, Beirut, Lebanon; ^14^School of Medicine and Medical Sciences, Holy Spirit University of Kaslik, Jounieh, Lebanon; ^15^College of Pharmacy, Gulf Medical University, Ajman, United Arab Emirates; ^16^Neurology Department, Henri Mondor Hospital, AP-HP, Créteil, France; ^17^INSERM U955-E01, IMRB, UPEC-Universite Paris-Est, Créteil, France

**Keywords:** stroke, knowledge of stroke, awareness of stroke, symptoms of stroke, Iraq stroke, Iraq, factors associated

## Abstract

**Introduction:**

Stroke is a highly prevalent condition with high rates of death and disability in Iraq and around the world. Knowledge of stroke and its associated risk factors is essential for disease prevention and rapid response when confronted with a stroke patient.

**Purpose:**

The purpose of this study is to assess stroke knowledge and identify factors associated with awareness among the Iraqi public.

**Material and methods:**

A questionnaire-based, cross-sectional survey was conducted on the Iraqi population. The self-administered online questionnaire contained three sections. The study got ethical approval from the Research Ethics Committee at the University of Baghdad.

**Results:**

The results showed that 26.8% of the participants reported knowledge regarding identifying all risk factors. In addition, 18.4% and 34.8% of the participants recognized all symptoms and mentioned all possible consequences of stroke, respectively. Previous medical history with chronic diseases had essential relationships with the response when faced with a person having an acute stroke. In addition, there was a significant relationship between gender, smoking history, and identification of early stroke symptoms.

**Conclusion:**

There was a lack of knowledge about risk factors for stroke among the participants. There is a need for an awareness program among the Iraqi people to raise their understanding of stroke that can reduce stroke mortality and morbidity.

## 1. Introduction

Globally, more than 12 million people suffer a stroke yearly, with an annual mortality rate above 5.5 million in the last two decades (1, 2). According to statistics, stroke is the second greatest cause of mortality and causes lasting disability in almost 5 million people (1, 3). As a result, it significantly increases the financial and social load on families and societies (2). Risk factors that affect stroke onset and progression include hypertension, diabetes mellitus, smoking, obesity, psychological factors, physical activity, and food. Approximately 80% of stroke cases can be halted if necessary precautions and appropriate measures are taken (2). Stroke risk is dramatically increasing more in emerging nations than in industrialized ones (2, 4, 5). In wealthy nations during the past four decades, stroke incidence has decreased by 42%, in contrast to developing (low- to middle-income) nations, where stroke rates have soared by 100%. (1). This change in stroke rates from industrialized to developing nations results from new risk factors, such as an unhealthy lifestyle, unbalanced diets, socioeconomic inequality, and a lack of access to the necessary treatment for individuals (1, 4).

Mortality from non-communicable diseases, including strokes, accounts for 55% of all deaths (6). In the context of Iraq, a country in the Middle East, coronary heart diseases and stroke are predominant diseases encountered in clinical practice. Incidence rates of stroke in Iraq ranged from 196.2 to 218.3 per 100,000 people in 2019, according to Global Burden of Disease 2019 Stroke Collaborators (2). Furthermore, 35.8% of Iraqis are estimated to suffer from hypertension, 14% from diabetes mellitus, 38% are smokers, and more than 30% are obese (6). Moreover, many Iraqi people have reported an unhealthy lifestyle, including a lack of physical activity and poor nutrition with high-calorie foods (7, 8). Therefore, several risk factors, including the high incidence of chronic diseases as well as the adoption of an unhealthy lifestyle, have been associated with the prevalence of stroke among the Iraqi population (9, 10). It is well-documented in the literature that the incidence of stroke can be prevented and reduced by knowing the risk factors that cause the disease, such as high blood pressure, smoking, and an unhealthy lifestyle (11–13). By identifying these factors, the initiation of preventive measures and raising awareness among the population can be achieved. In addition, complications from an acute stroke can be minimized by knowing what action is required for people who witness someone having a stroke (13, 14). Therefore, a careful assessment of the overall knowledge of stroke and its associated aggravating factors in the population is needed to assess the best preventive measure and implement community-oriented educational programs. Therefore, it is vital to explore features such as lifestyle, behavior, academic level, history of smoking, and socioeconomic status to understand the disparities in stroke knowledge between different sociodemographic groups.

Iraq's latest national epidemiological research on stroke is insufficient to determine the level of public knowledge. In order to reduce the burden of stroke, it is imperative to implement preventative measures, assess public knowledge and awareness of stroke and its risk factors, and improve patient knowledge and awareness through educational programs. Thus, this study aimed to evaluate stroke knowledge and determine the factors associated with stroke awareness among the Iraqi population.

## 2. Materials and methods

### 2.1. Study design

A cross-sectional observational study was conducted in Iraq between April and May 2021 from the general population using an anonymous survey. A Google forms-based electronic survey was produced and disseminated through social media platforms (i.e., WhatsApp and Facebook). Participants in this study had to be older than 18 to be eligible, and those with a stroke history were excluded. Participation was voluntary.

### 2.2. Minimal sample size

The target sample size was determined to be approximately 385 individuals based on another study's findings that approximately 71.8% of the participants were able to name at least three out of the five stroke risk factors (15) and because there were no comparable studies conducted in Iraq. Based on a confidence interval of 95%, a standard deviation of 0.5, and a margin of error of 5%, the Raosoft software sample size calculator calculated this amount as the minimum sample size required for an unlimited population size.

### 2.3. Questionnaire

The questionnaire was self-administered and required approximately 10 min to complete. The questionnaire was in Arabic, the native language of Iraqi people. The authors and five academic members reviewed the questionnaire, which then underwent a pilot test with five Iraqis in order to ensure the clarity of the questions. Next, the questions were modified based on their feedback.

The survey has been developed using the general principles of good survey design (16). Participants filled it out without the help of the researchers to avoid any potential influence when answering the questions. The current questionnaire, methods, and tools used in this study were mainly adapted from a study conducted in Jordan in which the general knowledge about stroke was assessed (17). The questionnaire was structured similarly to the one in Jordan in all the aspects that covered stroke knowledge except for the sociodemographic factors (i.e., economic status and residence area) because of the discrepancy between the two countries. However, differences were only in the sociodemographic characteristics due to the slight variation between the two countries. The questionnaire was structured to collect information about stroke in terms of symptoms, risk factors, early warning signs, and complications. Participants completed it without the assistance of investigators to avoid any potential influence when responding to questions. The opening section of the questionnaire covered the sociodemographic characteristics, including age, marital status, smoking status (positive when participant smoked for at least a year), employment status (employed vs. unemployed), monthly income, residence (urban vs. rural), educational level, and past medical history determined by self-report such as ever being diagnosed with a medical condition by a healthcare professional (e.g., hypertension, diabetes mellitus, dyslipidemia). Age was classified into four categories (18–29, 30–49, and above 50 years), while family income was divided into three categories: low (<400 K), intermediate (400–1,000 K), and high (>1,000 K) (18).

The second section evaluated the general knowledge related to stroke. Participants responded to whether the stroke is a disease that: (1) affects the brain, (2) is an old person's disease, (3) is contagious, (4) is hereditary, and (5) and can be prevented. Furthermore, this section assessed awareness of the risk factors of stroke, including old age, hypertension, diabetes mellitus, heart disease, high cholesterol, smoking, alcohol consumption, physical inactivity, obesity, and stress. This section also focused on participants' knowledge of early stroke warning signs, including (1) sudden numbness or weakness of the face, arms, or legs, (2) sudden difficulty speaking or understanding speech, (3) sudden blurry vision or visual impairment in one or both eyes, (4) sudden dizziness or loss of balance or coordination, and (5) sudden severe headache (18). In addition, participants reported potential consequences of stroke: (1) movement and functional problems (i.e., one-sided paralysis, loss of ability to walk, tiredness, fatigue), (2) cognitive and memory problems (i.e., loss of ability to speak, write, read, remember or understand), (3) visual problems (i.e., loss of sight or blurred vision), (4) emotional and personality changes (i.e., depression, anger, mood changes), and (5) long-term disabilities. Three questions assessed people's attitudes and reactions toward a patient experiencing stroke (e.g., willingness to take a patient to hospital care); two others were on curiosity and self-assessment, while the last one was to determine the sources of information of knowledge about stroke. Participants were given one point for each correct response to the earlier statements (19, 20). Missing answers were not counted. Sometimes, multiple answers were allowed, so the total score was higher than the total number of questions. The third section identified sources of information related to stroke among the participants.

### 2.4. Ethical approval

The study got ethical approval from the Research Ethics Committee at the University of Baghdad [SA 3/6502]. The study followed the Declaration of Helsinki's ethical standards and its later amendments or comparable ethical standards. All participants gave written informed consent to participate in the study.

### 2.5. Statistical analysis

Data collected were analyzed using the Statistical Package for Social Sciences (SPSS) version 25.0. Continuous variables were presented as mean ± standard deviation and 95% confidence interval. Categorical and ordinal variables were shown as frequencies (n) and percentages (%). Correlations between risk factors, early symptoms, and consequences of stroke with the socio-demographics and past medical history were determined by the Pearson chi-square or Fisher's exact test if the cell count was less than five. Logistic regression identified stroke risk factors, early symptoms, consequences, and response if faced with stroke as dependent variables and sociodemographic characteristics (gender, residence area, educational level, employment status, and smoking history) and health status as independent variables. Variables with a *p*-value of <0.2 in the bivariate analysis were included in the regression analysis (21). Potential confounders were eliminated if the *p*-value was >0.2 to protect against residual confounding. The results were presented as odds ratios (ORs) and 95% confidence interval. Statistical tests were two-tailed and indicated statistical significance at a *p*-value of < 0.05.

## 3. Results

### 3.1. Sociodemographic characteristics

A total of 609 participants completed the questionnaire. The majority of the participants lived in urban areas (93.6%), were female (78.3%), single (75.4%), and had a college degree (69.6%). The sociodemographic variables and health status of the participants are presented in Table 1. The most common comorbidities were heart disease (25.1%), followed by depression (22.6%) and obesity (16.1%). A family history of stroke was reported in 35.3% of the participants.

**Table 1:** Sociodemographic characteristics of the participants (*N* = 609).

**Variable**	***N* (%)**
**Age (in years)**	
<30	460 (75.5%)
30–49	119 (19.5%)
≥50	30 (5.0)
**Gender**	
Male	132 (21.7%)
Female	77 (78.3%)
**Residence area**	
Urban	570 (93.6%)
Rural	14 (2.3%)
**Education level**	
School (maximum 12)	73 (12.0%)
University degree	424 (69.6%)
Post-graduate degree	112 (18.4%)
**Marital status**	
Single	459 (75.4%)
Married	146 (24.0%)
Divorced or Widowed	4 (0.6%)
**Employment status**	
Unemployed	391 (64.2%)
Employed	218 (35.8%)
**Income level (Family income per month in Iraqi Dinar)**	
Low (< 400 K)	178 (29.2%)
Medium (400–1,000 K)	249 (40.9%)
High (>1,000 K)	182 (29.9%)
Family history of stroke	215 (35.3%)
**History of smoking (≥1 year)**	215 (35.3%)
**Health status**	
Heart diseases	153 (25.1%)
Depression	138 (22.7%)
Obesity	98 (16.1%)
Kidney disease	96 (15.8%)
Hypertension	85 (14.0%)
Peptic ulcer	72 (11.8%)
Dyslipidemia	71 (11.7%)
Diabetes mellitus	40 (6.6%)

### 3.2. Stroke knowledge

Familiarity with stroke was so high that 604 participants (99.2%) knew about it. In addition, most participants (76.2%) knew at least one person, a relative or friend, who had a stroke.

**Figure 1:**
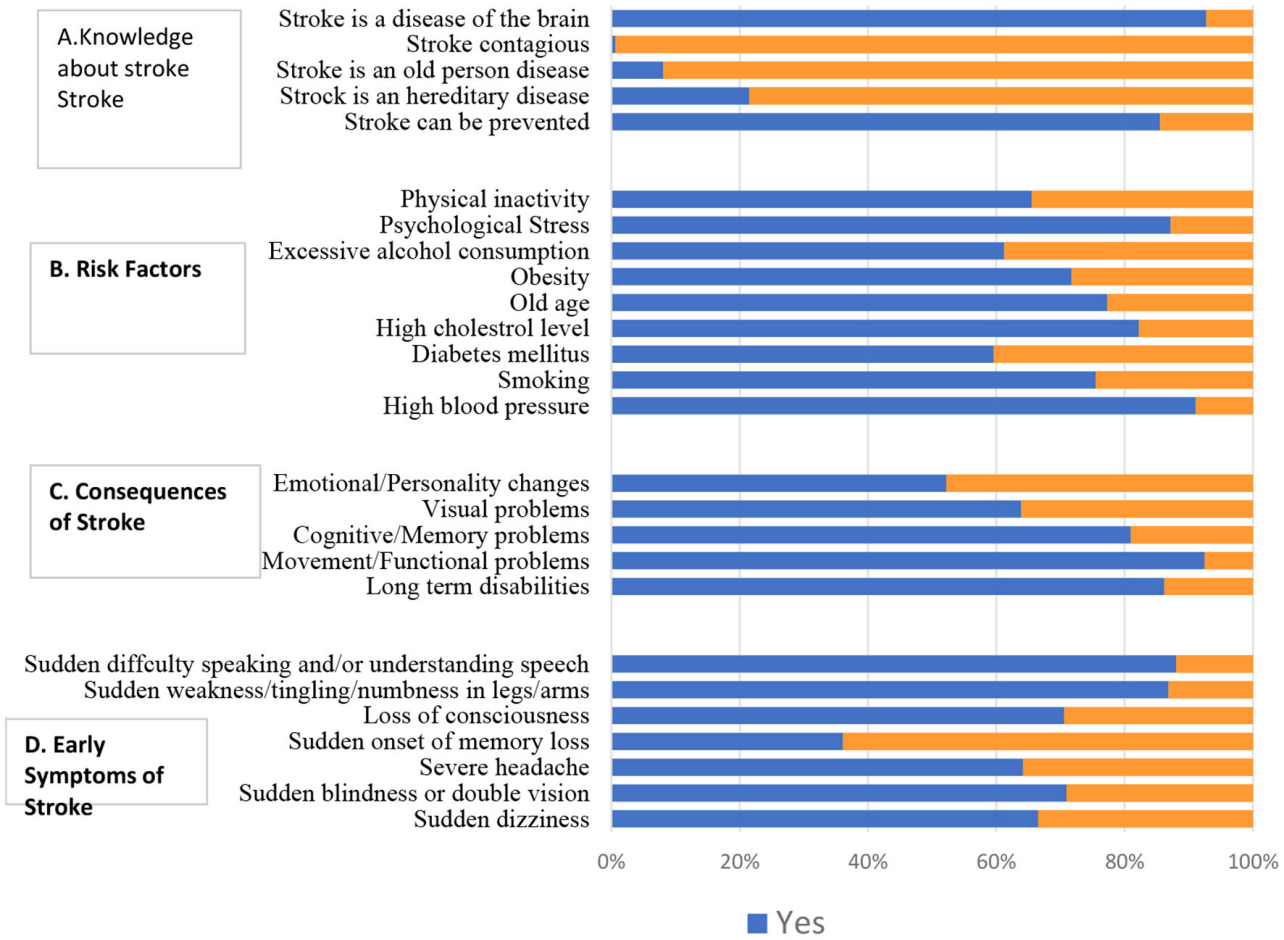
Knowledge among the participants (*n* = 609).

The level of knowledge about stroke is presented in Figure 1 and Table 2. Most of the sample were aware that stroke is a brain disease and can be prevented (92.8% and 85.6%, respectively) (Figure 1A). Furthermore, 91.1% believed that hypertension was the most common risk factor for stroke, followed by psychosocial stress (87.2%) and high cholesterol level (82.3%) (Figure 1B). The majority of participants stated that stroke could affect movement and functional problems (92.5%), lead to long-term disabilities (86.2%), and affect cognitive and memory problems (81.0%) (Figure 1C). The most common warning signs identified were “sudden difficulty in speaking or understanding speech” and “sudden weakness/numbness/tingling,” with 88.0% and 86.9%, respectively (Figure 1D).

**Table 2:** Number of risk factors, early symptoms, and consequences identified by the participants (*n* = 609).

**Variables**		**Frequency (%)**	**Cumulative, Frequency (%)**
Number of correct responses in the general knowledge	Less than two	8 (1.3)	8 (1.3)
	Two	84 (13.8)	92 (15.1)
	Three	389 (63.9)	481 (79.0)
	Four	119 (19.5)	600 (98.5)
	Five	9 (1.5)	609 (100)
Number of risk factors identified	Zero	10 (1.6)	10 (1.6)
	One	6 (1.0)	16 (2.6)
	Two	10 (1.6)	26 (4.3)
	Three	24 (3.9)	50 (8.2)
	Four	43 (7.1)	93 (15.3)
	Five	64 (10.5)	157 (25.8)
	Six	82 (13.5)	239 (39.2)
	Seven	115 (18.9)	354 (58.1)
	Eight	92 (15.1)	446 (73.2)
	Nine	163 (26.8)	609 (100)
Number of early symptoms identified	Zero	27 (4.4)	27 (4.4)
	One	8 (1.3)	35 (5.7)
	Two	32 (5.3)	67 (11.0)
	Three	50 (8.2)	117 (19.2)
	Four	92 (15.1)	209 (34.3)
	Five	159 (26.1)	368 (60.4)
	Six	129 (21.2)	497 (81.6)
	Seven	112 (18.4)	609 (100)
Number of consequences identified	Zero	26 (4.3)	26 (4.3)
	One	12 (2.0)	38 (6.2)
	Two	43 (7.1)	81 (13.3)
	Three	134 (22.0)	215 (35.3)
	Four	182 (29.9)	397 (65.2)
	Five	212 (34.8)	609 (100)

**Figure 2:**
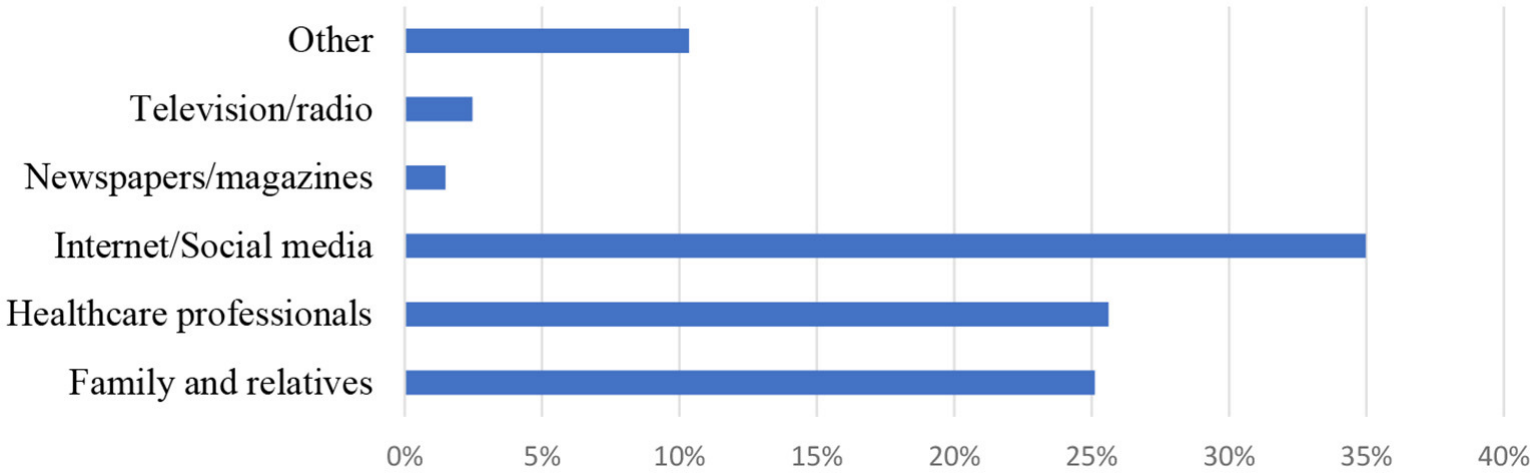
Sources of information (%) related to stroke among the participants (*n* = 609).

Regarding identifying stroke risk factors, only 26.8% identified all risk factors, 18.4% of the participants identified all symptoms, and 34.8% reported all possible consequences of stroke. The primary sources of information were the Internet/social media (35.0%), healthcare professionals (25.6%), and family/relatives (25.1%) (Figure 2).

About a quarter of participants (26.6%) believed that they did not know stroke's effects well. However, the majority of the participants (91.1%) were curious to have more information related to stroke, including symptoms, emergency signs, and response when facing someone with a stroke attack and the consequences of the stroke. In addition, most participants (97.2%) believed that the role of the family is essential in providing care to a patient with a stroke at an early stage. In addition, more than three-quarters of participants (77.8%) believed that stroke disease could make patients' lives unhappy.

### 3.3. Bivariate analysis

In the bivariate analysis, a significantly higher proportion of female participants than male participants (97.1% vs. 90.1%) recognized at least one warning symptom of stroke. Furthermore, the participants without a history of smoking vs. a positive history of smoking (96.7% vs. 88.4%) recognized at least one warning symptom of stroke. A significantly higher proportion of participants without a history of diabetes mellitus compared to patients diagnosed with diabetes (96.4% vs. 87.5%) correctly identified the consequences emerging from stroke (Table 3).

**Table 3:** Association of risk factors, early symptoms, and consequences of stroke with the sociodemographic variables and health status (*n* = 609).

**Variables**		**Risk factor(s) identified (**≥**1)**			**Early symptom(s) identified (**≥**1)**			**Consequence(s) identified (**≥**1)**		
		**Yes (*n* = 599), n (%)**	**No (*n* = 10), n (%)**	***P*-value**	**Yes (*n* = 582), n (%)**	**No (*n* = 27), n (%)**	***P*-value**	**Yes (*n* = 583), n (%)**	**No (*n* = 26), n (%)**	***P*-value**
**Sociodemographic variables**										
Gender	Male	128 (97.0)	4 (3.0)	0.156	119 (90.2)	13 (9.8)	**0.001**	125 (94.7)	7 (5.3)	0.507
	Female	471 (98.7)	6 (1.3)		463 (97.1)	14 (2.9)		458 (96.0)	19 (4.0)	
Age (years)	<30	454 (98.7)	6 (1.3)	0.081	442 (96.1)	18 (3.9)	0.396	439 (95.4)	21 (4.6)	0.818
	30–49	117 (98.3)	2 (1.7)		111 (93.3)	8 (6.7)		115 (96.6)	4 (3.4)	
	≥50	28 (93.3)	2 (6.7)		29 (96.7)	1 (3.3)		29 (96.7)	1 (3.3)	
Residence area	Urban	560 (98.2)	10 (1.8)	0.706	545 (95.6)	25 (4.4)	0.496	544 (95.4)	26 (4.6)	0.395
	Rural	14 (100)	0 (0)		14 (100)	0 (0)		14 (100)	0 (0)	
	Urban-rural	25 (100)	0 (0)		23 (92.0)	2 (8.0)		25 (100)	0 (0)	
Marital status	Single/divorced/widowed	456 (98.5)	7 (1.5)	0.653	445 (96.1)	18 (3.9)	0.244	442 (95.5)	21 (4.5)	0.563
	Married	143 (97.9)	3 (2.1)		137 (93.8)	9 (6.2)		141 (96.6)	5 (3.4)	
Educational level	School	71 (97.3)	2 (2.7)	0.431	69 (94.5)	4 (5.5)	0.644	68 (93.2)	5 (6.8)	0.245
	University	528 (98.5)	8 (1.5)		513 (95.7)	23 (4.3)		515 (96.1)	21 (3.9)	
Employment status	Unemployed	383 (98.0)	8 (2.0)	0.293	375 (95.9)	16 (4.1)	0.584	373 (95.4)	18 (4.6)	0.585
	Employed	216 (99.1)	2 (0.9)		207 (95.0)	11 (5.0)		210 (96.3)	8 (3.7)	
Income level	Low	175 (98.3)	3 (1.7)	0.724	170 (95.5)	8 (4.5)	0.897	168 (94.4)	10 (5.6)	0.567
	Medium	246 (98.8)	3 (1.2)		239 (96.0)	10 (4.0)		240 (96.4)	9 (3.6)	
	High	178 (97.8)	4 (2.2)		173 (95.1)	9 (4.9)		175 (96.2)	7 (3.8)	
History of smoking (≥1 year)	No	516 (98.7)	7 (1.3)	0.146	506 (96.7)	17 (3.3)	**< 0.001**	501 (95.8)	22 (4.2)	0.850
	Yes	83 (96.5)	3 (3.5)		76 (88.4)	10 (11.6)		82 (95.3)	4 (4.7)	
**Health Status**										
Hypertension	No	516 (98.5)	8 (1.5)	0.578	504 (96.2)	20 (3.8)	0.066	504 (96.2)	20 (3.8)	0.170
	Yes	83 (97.6)	2 (2.4)		78 (91.8)	7 (8.2)		79 (92.9)	6 (7.1)	
Diabetes mellitus	No	561 (98.6)	8 (1.4)	0.084	546 (96.0)	23 (4.0)	0.077	548 (96.3)	21 (3.7)	**0.008**
	Yes	38 (95.0)	2 (5.0)		36 (90.0)	4 (10.0)		35 (87.5)	5 (12.5)	
Dyslipidaemia	No	531 (98.7)	7 (1.3)	0.068	516 (95.9)	22 (4.1)	0.256	518 (96.3)	20 (3.7)	0.064
	Yes	68 (95.8)	3 (4.2)		66 (93.0)	5 (7.0)		65 (91.5)	6 (8.5)	
Heart diseases	No	451 (98.9)	5 (1.1)	0.067	436 (95.6)	20 (4.4)	0.922	436 (95.6)	20 (4.4)	0.806
	Yes	148 (96.7)	5 (3.3)		146 (95.4)	7 (4.6)		147 (96.1)	6 (3.9)	
Kidney disease	No	504 (98.2)	9 (1.8)	0.614	489 (95.3)	24 (4.7)	0.497	492 (95.9)	21 (4.1)	0.620
	Yes	95 (99.0)	1 (1.0)		93 (96.9)	3 (3.1)		91 (94.8)	5 (5.2)	
Peptic ulcer	No	527 (98.1)	10 (1.9)	0.243	514 (95.7)	23 (4.3)	0.622	514 (95.7)	23 (4.3)	0.963
	Yes	72 (100)	0 (0)		68 (94.4)	4 (5.6)		69 (95.8)	3 (4.2)	
Depression	No	464 (98.5)	7 (1.5)	0.576	449 (95.3)	22 (4.7)	0.599	450 (95.5)	21 (4.5)	0.669
	Yes	135 (97.8)	3 (2.2)		133 (96.4)	5 (3.6)		133 (96.4)	5 (3.6)	
Obesity	No	504 (98.6)	7 (1.4)	0.227	490 (95.9)	21 (4.1)	0.375	490 (95.9)	21 (4.1)	0.656
	Yes	95 (96.9)	3 (3.1)		92 (93.9)	6 (6.1)		93 (94.9)	5 (5.1)	

Numbers in bold indicate significant *p*-values.

### 3.4. Response to somebody with symptoms of stroke

The majority of participants (88.7%) reported that their first action in response to witnessing a patient with stroke symptoms was to take the patient directly to a hospital; the reset was either calling the doctor (5.42%) or not knowing what to do 5.91%. Regarding the response in case of facing somebody with acute stroke symptoms by taking them to the hospital, a significantly higher proportion of single participants compared to married responded by taking the patient to the hospital (90.3% vs. 83.6%, *p* = 0.026). Furthermore, the number of correct answers was associated with people with no history of hypertension compared to those having hypertension (90.3% vs. 78.8%, *p* = 0.002), people with no history of diabetes compared to those having diabetes (90.0% vs. 70.0%, *p* < 0.001), and people with no history of dyslipidemia compared to having dyslipidemia (89.6% vs. 81.7%, *p* = 0.048) (Table 4).

**Table 4:** Association of response in case of facing somebody with acute symptoms of a stroke (identified by taking the patient to the hospital) and with sociodemographic variables and health status of the participants (*n* = 609).

**Variables**		**Response in case of facing somebody with acute symptoms of stroke identified by taking the patient to the hospital**		
		**Yes (*****n*** = **540)**, ***n*** **(%)**	**No (*****n*** = **69)**, ***n*** **(%)**	***P*** **-value**
**Sociodemographic variables**				
Gender	Male	118 (89.4)	14 (10.6)	0.767
	Female	422 (88.5)	55 (11.5)	
Age (years)	<30	414 (90.0)	46 (10.0)	0.176
	30–49	100 (84.0)	19 (16.0)	
	≥50	26 (86.7)	4 (13.3)	
Residence area	Urban	505 (88.6)	65 (11.4)	0.818
	Rural	12 (85.7)	2 (14.3)	
	Urban-Rural	23 (92.0)	2 (8.0)	
Marital status	Single/divorced/widowed	418 (90.3)	45 (9.7)	**0.026**
	Married	122 (83.6)	24 (16.4)	
Educational level	School	65 (89.0)	8 (11.0)	0.915
	University	475 (88.6)	61 (11.4)	
Employment status	Unemployed	351 (89.8)	40 (10.2)	0.251
	Employed	189 (86.7)	29 (13.3)	
Income level	Low	154 (86.5)	24 (13.5)	0.366
	Medium	226 (90.8)	23 (9.2)	
	High	160 (87.9)	22 (12.1)	
History of smoking (≥1 year)	No	467 (89.3)	56 (10.7)	0.232
	Yes	73 (84.9)	13 (15.1)	
**Health status**				
Hypertension	No	473 (90.3)	51 (9.7)	**0.002**
	Yes	67 (78.8)	18 (21.2)	
Diabetes mellitus	No	512 (90.0)	57 (10.0)	**< 0.001**
	Yes	28 (70.0)	12 (30.0)	
Dyslipidaemia	No	482 (89.6)	56 (10.4)	**0.048**
	Yes	58 (81.7)	13 (18.3)	
Heart diseases	No	410 (89.9)	46 (10.1)	0.095
	Yes	130 (85.0)	23 (15.0)	
Kidney disease	No	457 (89.1)	56 (10.9)	0.456
	Yes	83 (86.5)	13 (13.5)	
Peptic ulcer	No	480 (89.4)	57 (10.6)	0.128
	Yes	60 (83.3)	12 (16.7)	
Depression	No	424 (90.0)	47 (10.0)	0.052
	Yes	116 (84.1)	22 (15.9)	
Obesity	No	458 (89.6)	53 (10.4)	0.088
	Yes	82 (83.7)	16 (16.3)	

Numbers in bold indicate significant *p*-values.

### 3.5. Multivariable analysis

The multivariable analysis demonstrated that female participants were more likely than male participants to identify a risk factor when recognizing at least one risk factor as the dependent variable (OR = 2.5, *p*-value of 0.047). Moreover, smokers were less likely to name a risk factor than non-smokers (OR = 0.38, *p*-value of 0.044). Diabetic patients exhibited significantly lower odds of recognizing stroke symptoms than non-diabetic patients (OR = 0.35), with a *p*-value of 0.024 when the response to stroke symptoms discovered through bringing the patient to the hospital was the dependent variable (Table 5).

**Table 5:** Multivariate analysis of associations between sociodemographic data of the participants (*n* = 609) and identification of risk factors, early symptoms, consequences of stroke, and taking a patient to a hospital.

**Variables**	**β (SE)**	**OR (95% CI)**	***P*-value**
**Risk factor(s) identified (**≥**1)**			
Age (between 30 and 49 vs < 30^*^)	−0.57 (1.02)	0.56 (0.07–4.18)	0.57
Age (≥50 vs. < 30^*^)	−1.63 (1.22)	0.19 (0.18–2.12)	0.17
Marital status (Married vs. single^*^)	0.95 (1.07)	2.60 (0.31–21.45)	0.37
Gender (Female vs. male^*^)	0.53 (0.80)	1.69 (0.35–8.19)	0.50
Smoker (Yes vs. No^*^)	−0.70 (0.83)	0.49 (0.09–2.54)	0.40
Diabetes (Yes vs. No^*^)	−0.68 (0.99)	0.50 (0.07–2.54)	0.49
Dyslipidaemia (Yes vs. No^*^)	−0.21 (0.91)	0.81 (0.13–4.85)	0.81
Heart diseases (Yes vs. No^*^)	−0.82 (0.71)	0.43 (0.10–1.76)	0.24
**Early symptom(s) identified (**≥**1)**			
Gender (Female vs. male^*^)	0.92 (0.46)	2.50 (1.01–6.21)	**0.047**
Marital status (Married vs. single^*^)	0.09 (0.46)	1.10 (0.44–2.72)	0.835
Smoker (Yes vs. No^*^)	−0.94 (0.46)	0.38 (0.15–0.97)	**0.044**
Hypertension (Yes vs. No^*^)	−0.52 (0.51)	0.59 (0.21–1.63)	0.315
Diabetes (Yes vs. No^*^)	−0.63 (0.66)	0.52 (0.14–1.92)	0.334
**Consequence(s) identified (**≥**1)**			
Marital status (Married vs. single^*^)	0.57 (0.53)	1.77 (0.62–5.01)	0.283
Hypertension (Yes vs. No^*^)	−0.04 (0.63)	0.96 (0.27–3.36)	0.952
Diabetes (Yes vs. No^*^)	−1.17 (0.66)	0.30 (0.08–1.13)	0.077
Dyslipidaemia (Yes vs. No^*^)	−0.50 (0.63)	0.60 (0.17–2.11)	0.430
**Taking a patient to a hospital**			
Hypertension (Yes vs. No^*^)	−0.46 (0.38)	0.62 (0.29–1.32)	0.219
Diabetes (Yes vs. No^*^)	−1.02 (0.45)	0.35 (0.14–0.87)	**0.024**
Dyslipidaemia (Yes vs. No^*^)	0.29 (0.48)	1.34 (0.52–3.45)	0.541
Age (between 30 and 49 vs. < 30^*^)	−0.14 (0.41)	0.86 (0.38–1.96)	0.728
Age (≥50 vs. < 30^*^)	0.26 (0.68)	1.30 (0.34–4.96)	0.692
Marital status (Married vs. single^*^)	−0.46 (0.39)	0.63 (0.29–1.37)	0.247
Heart diseases (Yes vs. No^*^)	−0.14 (0.31)	0.86 (0.47–1.59)	0.646
Peptic ulcer (Yes vs. No^*^)	−0.05 (0.39)	0.95 (0.43–2.05)	0.898
Depression (Yes vs No^*^)	−0.45 (0.31)	0.63 (0.34–1.18)	0.154
Obesity (Yes vs. No^*^)	−0.12 (0.37)	0.88 (0.43–1.82)	0.748

β, Beta; SE, standard error; OR; adjusted ratio; CI, confidence interval. Numbers in bold indicate significant *p*-values.

Logistic regression by taking the identification of stroke risk factors, stroke early symptoms, stroke consequences, and response if faced with stroke as the dependent variables and sociodemographic factors (gender, residence area, educational level, employment status, and smoking history) and health status as independent variables.

* Stands for reference category.

## 4. Discussion

The study evaluated knowledge of stroke among a sample of the general Iraqi community. Nearly every participant had heard about stroke. Nonetheless, the findings revealed that 21.0% of participants had 75% of the right answers concerning stroke, 60.8% had identified risk factors, 39.6% had identified early symptoms, and 64.5% had recognized the effects of the disease. The percentages of correct responses, however, are higher than those seen in numerous other studies conducted internationally among the general population (22–27), indicating that more than half of the sample may be able to identify at least one stroke risk factor. The high awareness toward stroke and its associated elements in our study may be explained by the fact that the majority of the study participants are young and have high level of education.

The results revealed that hypertension and psychological stress were the most well-known risk factors for stroke in nearly 90% of the participants. This is higher than results reported in other studies on the general population conducted in several countries (25, 28, 29). However, similarities were found in other studies conducted in Jordan and Lebanon (17, 19). Although diabetes is the most well-known risk factor for stroke, it was the lowest reported among the study participants (<60%). Similarly, another study found that approximately three-quarters of the sample did not identify diabetes as a risk factor for stroke (25). In the current study, the majority of participants (88.9%) understood the significance of getting to the hospital as soon as possible after a stroke was identified, which is consistent with other published findings demonstrating that a high percentage of participants understood the need for immediate medical care (2–4, 27). A key tactic in reducing mortality, enhancing prognosis, and limiting long-term sequelae is raising general public knowledge of the significance of early hospital presentation.

The results showed that female participants had better knowledge about the early symptom(s) identified (≥1) than male participants. However, several studies did not detect gender differences in the early symptom(s) identified (5, 27). Women tend to be more knowledgeable and might have a greater interest in health topics than men and take more time to seek information (6). Moreover, non-smokers outperformed smokers in terms of their understanding of early-detected symptoms (1). Yet, among study participants, there was no correlation with any other kind of knowledge. Diabetes patients showed higher awareness of the known effects of stroke and improved responsiveness when a stroke victim displayed symptoms by bringing the patient to the hospital. Furthermore, married participants and individuals with hypertension or dyslipidemia reported knowing this last statement better. There may be a strong indicator that individuals with chronic illnesses are more knowledgeable about strokes. However, the percentage of knowledgeable subjects was low because of other important factors, such as failure to recognize stroke warning symptoms, which sometimes induced hospital presentation delays and worsened stroke effects (6).

In light of these findings, awareness programs are required for the general Iraqi population, targeting people at particular risk of stroke. These programs should include educating people regarding the focus on the association of symptoms of stroke, the potential severity of the disease, and how avoiding delays in visiting the hospital correlates with a more favorable prognosis.

### 4.1. Clinical implications

The study added interesting data about stroke disease awareness to the literature, highlighting the lack of knowledge regarding stroke and related issues among the Iraqi population. Several factors influenced knowledge about stroke among Iraqi participants, such as previous medical history with chronic diseases, marital status, gender, and history of smoking.

### 4.2. Limitations

This study has several limitations. The results could not be representative of the entire Iraqi population as the majority of participants were well-educated with computer literacy and internet access; thus, less-educated people and those who did not have access to the internet were not assessed. Thus, selection bias plays a role in our study because the study targeted educated urban people and involved snowball sampling. Additionally, its cross-sectional design cannot infer causality. Information bias could also exist as the study questionnaire was online and answers were self-reported. The answers to stroke awareness might be overestimated because the questionnaire used consisted of multiple-choice questions with limited options available; thus, the participants could have guessed the answers.

## 5. Conclusion

There was a lack of knowledge of the risk factors for stroke among the Iraqi participants. Health status with chronic disease, female gender, married status, and smoking history was associated with good knowledge to identify some stroke-related issues. Knowledge and awareness among the Iraqi people regarding stroke risk factors, symptoms, complications, and actions to treat stroke patients should be addressed as an essential effect of reducing stroke mortality and morbidity.

## Data availability statement

The raw data supporting the conclusions of this article will be made available by the authors, without undue reservation.

## Ethics statement

The studies involving human participants were reviewed and approved by the Research Ethics Committee at the University of Baghdad [SA 3/6502]. The patients/participants provided their written informed consent to participate in this study.

## Author contributions

HA-O, FJ, DM, and SH: conceptualization. HA-O and ZaK: project administration. SH and CH: formal analysis. DM: investigation. HA-O, FJ, DM, and HH: methodology. HA-O, ZaK, FJ, MB, HA, ZeK, YA, CH, and SM: writing the original draft. DM, SH, and HH: reviewing and editing. All authors read and approved the final manuscript.
